# Species Occurrence and Seasonal Variation of Malaria Vectors in Hadiya Zone, Ethiopia

**DOI:** 10.1155/bmri/4553611

**Published:** 2026-02-18

**Authors:** Anmut Assemie, Dasash Mulu, Eyerus Mekuriaw, Workineh Muluken

**Affiliations:** ^1^ Department of Biology, Wachemo University, Hossana, Ethiopia, wcu.edu.et; ^2^ Department of Statistics, Wachemo University, Hossana, Ethiopia, wcu.edu.et

**Keywords:** breeding sites, characterization, malaria vector, occurrence, variation

## Abstract

Environmental change can alter the species occurrence and seasonal distribution of malaria vectors to higher altitudes and latitudes. Highlands remain dynamic due to factors that favor their growth and development. Invasive species have proliferated into new ecological niches, increased. These studies aimed to determine the species occurrence and seasonal variation of malaria vectors in the selected study area. Entomological surveys were conducted in different types of larval habitats from October 2023 up to June 2024 within four purposively selected study villages. The species were morphologically identified using a stereomicroscope, and then data was analyzed using R version 4.3.1 (2023‐06‐16 ucrt) statistical analysis software. A total of 721 malaria vector larvae were collected, representing *Anopheles gambiae* s.l., *An. funestus* s.l., *An. coustani*, and *An. pharoensis*. *An. gambiae* s.l. was the dominant species, accounting for 43.82% (*n* = 316) of all collections, while *An. pharoensis* was the least abundant (7.9%, *n* = 57). Spatial variation was observed, with Shemo Boyo recording the highest number of larvae (45.50%, *n* = 328), whereas Kemecho Borara had the lowest (9.57%, *n* = 69). Among the habitat types, ditches had the highest mean larval density (2.61 larvae per sample), followed by swamps (1.5) and riverbeds (0.8), whereas water pans had the lowest density (0.14). Overall mean larval density was 1.11 larvae per sample, and larval abundance significantly differed across habitat categories (f (3,647) = 4.005, *p* = 0.012). These findings indicate that *An. gambiae* s.l. is the predominant malaria vector in the area and likely plays a primary role in local transmission. Further studies on spatial mapping, physicochemical characterization, habitat preference, and isolation of malaria parasites are recommended to guide targeted larval source management and reduce the burden of mosquito‐borne diseases.

## 1. Introduction

In the world, over 3500 mosquitoes have been recorded [1]. Of the roughly 537 *Anopheles* species [2], only 70–80 have been documented as vectors of human malaria globally [3]. Of these, 41 species are recognized as primary malaria vectors [4, 5] with a high capacity for malaria transmission. In Africa, the *Anopheles gambiae* sensu lato (s.l.) complex and the *Anopheles funestus* group include the most important malaria vectors. The *An. gambiae* s.l. complex comprises eight sibling species: *An. gambiae* sensu stricto, *An. coluzzii*, *An. arabiensis*, *An. melas*, *An. merus*, *An. bwambae*, and *An. quadriannulatus* [6–8], and *An. amharicus* [8].

The most efficient malaria vectors in Africa are *An. gambiae* s.s. and *An. funestus* [9, 10], due to their strong anthropophagy, or preference for feeding on human blood [11–18]. In contrast, *An. arabiensis* is generally considered a less efficient vector, as it exhibits greater flexibility in host selection [19].

According to Adugna et al. [20], a total of 110,305 *Anopheles* mosquitoes were collected across various regions of Ethiopia, representing 35 species. *An. arabiensis* was the most abundant, while *An. maculipalpis* and *An. wilsonii* were the least common. The highest species abundance was recorded in central Ethiopia, whereas the lowest was observed in the eastern region. The second, third, and fourth most abundant species were predominantly collected from the southern, northern, and western parts of the country, respectively.

In some areas, *An. pharoensis*, *An. funestus*, and *An. nili* are known malaria vectors [21]. In addition, *An. coustaini*, *An. pauraludis*, *An. ziemanni*, and *An. d’thalia* have been recorded as having vector potential [22]. In the Gambella region, at least eight Anopheline species, each differing in their habitats, were found to be prevalent. These species are the following: *An. gambiae*, *An. pharoensis*, *An. funestus*, *An. nili*, *An. coustaini*, *An. pauraludis*, *An. ziemanni*, and *An. squamosus* [23].

*Anopheles* mosquitoes serve as principal vectors of malaria and are implicated in the transmission of several other vector‐borne diseases, including filariasis, dengue, yellow fever, chikungunya, and Japanese encephalitis. They continue to drive substantial morbidity and mortality, particularly across tropical and subtropical regions [24]. Malaria is a vector‐borne disease transmitted by infective female Anopheles mosquito bites [10, 25, 26]. Malaria affects more than 97 countries, putting approximately 44% of the global population at risk. It is responsible for an estimated 216 million cases and 445,000 deaths annually, with over 90% of these occurring in the African region. Furthermore, 14 countries in sub‐Saharan Africa, along with India, account for 80% of the global malaria burden [27]. In southern Ethiopia, including the Hadiya Zone, the pooled prevalence of malaria has been estimated at 19.19%, largely due to the presence of efficient malaria vectors [28]. However, data on the species occurrence and seasonal variation of these vectors in the region remain limited.

Previous studies in southwestern Ethiopia reported that *An. arabiensis* and *An. funestus* were the dominant species, with their abundance and biting activity showing marked seasonal peaks associated with rainfall patterns. However, vector composition and dynamics can vary considerably across ecological zones. Since no similar study had been conducted in the Hadiya Zone, where environmental and altitudinal factors differ significantly from the Southwestern lowlands [29]. Therefore, the present study aimed to generate updated data on the species occurrence and seasonal variation of malaria vectors under these distinct ecological and climatic conditions.

## 2. Materials and Methods

### 2.1. Description of the Study Area

**Figure 1:**
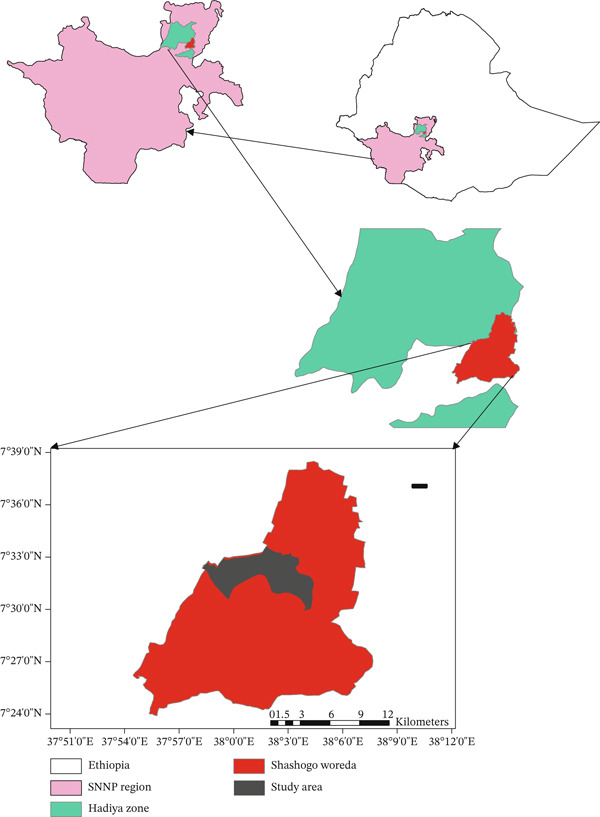
Map of study area.

The present study was conducted in the Hadiya Zone, located in the Southern region of Ethiopia. The zone is bordered to the south by Kembata Tembaro, Southwest by the Dawro Zone, West by the Omo River, which separates it from the Oromia Region, and the Yem Special Woreda, North by Gurage, Northeast by Silte, and East by the Alaba Special Woreda. The woredas of Mirab Badawacho and Misraq Badawacho form an exclave, separated from the rest of the zone by Kembata Tembaro. Hadiya Zone lies approximately 235 km from Addis Ababa, between 7°39 ^′^60″ N latitude and 37°44 ^′^60″ E longitude (Figure 1).

### 2.2. Study Design and Sampling Methods

A longitudinal study was conducted to generate baseline data, with follow‐up surveys carried out to assess the occurrence and seasonal variation of malaria vectors. Entomological surveys were conducted in the most representative villages of Hadiya zone. The study villages were selected based on altitude and temperature, because the sites which were located between 1250 and 2500 m above sea level, temperature between 21°C and 29°C, and an altitude defined as a lowland area are malarias.

### 2.3. Rearing of Malaria Vectors and Species Identification

Larvae of malaria vectors were collected from selected breeding sites in Hadiya Zone, central Ethiopia, with sampling conducted twice monthly between 09:00 and 16:00 h. Each breeding site type was recorded as positive or negative once per survey. A maximum of 10 dips was taken per site type during each survey; when individual pools were too small for multiple dips, a single dip was taken, and additional pools of the same type were sampled until 10 dips were achieved. Thus, 10 dips were effectively collected from 10 distinct pools of each breeding site type per survey.

Then, the collected larvae were reared in the biology laboratory at room temperature and 75%–85% RH. During rearing, the larvae were fed continuous access to a 10% sugar solution. After pupation, pupae were transferred to mosquito cages until they emerged as adults.

According to Gillies and De Meillon [30], the development time of malaria vector larvae typically ranges from 7 to 12 days, depending on factors such as temperature, larval density, food availability, and the specific strain. Pupae subsequently transform into adults within 2 to 4 days, depending on environmental conditions. Adult malaria vectors are then identified morphologically using the keys of Gillies and Coetzee after being killed with chloroform [31].

### 2.4. Data Analysis

Malaria vector data were entered into Microsoft Excel for cleaning and subsequently analyzed using R version 4.3.1 (2023‐06‐16 ucrt) statistical software. Data consistency and completeness were checked at the field level, and data coding and entry were performed to assess the monthly variation of malaria vectors in the study area. Differences in mean abundance between species and among villages were analyzed using one‐way analysis of variance (ANOVA) with a significance level of *p* < 0.05. Simple descriptive statistics, including counts, percentages, and tables, were used to summarize and present the variables.

## 3. Results

### 3.1. Occurrence of Malaria Vectors

A total of 1216 mosquito species were collected from the selected study area during the study season. From these, 721 (59.29%) belong to the genus Anopheles (malaria vector). Based on morphological identification, four malaria vector species were recorded, which belonged to *An. gambiae* s.l (43.82%), *An. funestus* group (29.4%), *An. coustani* (18.86%), and *An. pharoensis* (7.9%) (Table 1).

**Table 1:** The occurrence of malaria vectors in the study area.

Species identified	Occurrence (N)	%
Anopheles gambiae s.l	316	43.82
Anopheles funestus s.l	212	29.4
Anopheles coustani	136	18.86
Anopheles pharoensis	57	7.9
Total	721	100

### 3.2. Seasonal Variation of Malaria Vectors

The highest malaria vectors (45.50%) were collected from Shemo Boyo village dominated by *An. gambiae* s.l, (43.83%, *n* = 316) while the lowest (9.57%, *n* = 69) was in Kemecho Borara, which was dominated by *An. funestus s.l* (66.66%, *n* = 46) species. In Kemecho Borara, a total of 69 (9.57%) species was collected, which belongs to *An. gambiae* s.l (33.33%, *n* = 23) and *An. funestus s.l* (66.66%, *n* = 46) only (Table 2).

**Table 2:** Distribution of malaria vectors in the study villages.

Study sites	Malaria vectors (*N* %)				Total (*N* %)	*f* value	*p* value
	*An. gambiae s.l*	*An. funestus s.l*	*An. coustani*	*An. pharoensis*			
Kemecho Borara	23 (33.33)	46 (66.66)	0 (0.00)	0 (0.00)	69 (9.57)	3.12	0.058
Musagesa Hablera	54(41.53)	49(37.69)	27(20.76)	0(0.00)	130(18.03)		
Ajacho	112 (57.73)	28 (14.43)	35 (18.04)	19 (9.79)	194 (26.91)		
Shemo Boyo	127 (38.72)	89 (27.13)	74 (22.56)	38 (11.58)	328 (45.50)		
Total	316 (43.83)	212 (29.40)	136 (18.86)	57 (0.91)	721 (100)		

In all study seasons, 721 malaria vectors were collected during the study seasons. At the end of the rainy season, 528 (73.23%) malaria vectors were collected from October to January. However, the population of malaria vectors decreased to 193 (26.768%) in February, March, April, May, and June, coinciding with the dry season, as indicated in Table 3.

**Table 3:** Seasonal variations of malaria vectors during study season.

Study periods	Malaria vector species (*N* %)				Total (*N* %)
	*An. gambiae s.l*	*An. funestus s.l*	*An. coustani*	*An. pharoensis*	
October	97 (47.78)	53 (26.11)	36 (17.73)	17 (8.37)	203 (28.16)
November	109 (49.77)	61 (27.85)	41 (18.72)	8 (3.65)	219 (30.37)
December	16 (33.33)	29 (60.41)	0 (0.00)	3 (6.25)	48 (6.65)
January	13 (22.41)	18 (31.03)	27 (46.55)	0 (0.00)	58 (8.04)
February	0(0.00)	0 (0.00)	16 (100)	0 (0.00)	16 (2.22)
March	0 (0.00)	0 (0.00)	5 (100)	0 (0.00)	5 (0.69)
April	0 (0.00)	8 (100)	0 (0.00)	0 (0.00)	8 (1.11)
May	32 (47.76)	27 (40.29)	0 (0.00)	8 (11.94)	67 (9.30)
June	49 (50.51)	16 (16.49)	11 (11.34)	21 (21.65)	97 (13.45)
Total	316 (43.83%)	212 (29.40%)	136 (18.86%)	57 (0.91%)	721(100)

### 3.3. Larval Habitat Preference

The malaria vector habitats were characterized in the selected study villages during data collection. A total number of 53 different larval habitats were sampled, which belonged to four major types of habitats, such as ditch, river‐bed, water pan, and swamp, for different study periods.

The highest larvae per sample of malaria vector species (2.61) was recorded in the ditch, followed by the swamp (1.5) and the seasonal river bed (0.8 per sample). The lowest mean number of malaria vector larvae (0.14) was recorded in the water pan. The distribution of larvae with the breeding sites was statistically significant at *p* = 0.012 as shown in Table 4.

**Table 4:** Distribution of malaria vector larvae across different breeding habitat types.

Habitat types	No. of samples	*Number of* malaria vectors per sample (mean)				Total	*f* value	*p* value
		*An. gambiae s.l*	*An. funestus s.l*	*An. coustani*	*An. pharoensis*			
Ditch	137	73 (0.53)	173 (1.26)	91 (0.66)	21 (0.15)	358 (2.61)	4.005	0.012
River‐bed	273	167 (0.61)	11 (0.04)	32 (0.18)	9 (0.03)	219 (0.80)		
Water pan	162	17 (0.15)	0 (0.00)	5 (0.03)	0 (0.00)	22 (0.14)		
Swamp	79	59 (0.75)	28 (0.35)	8 (0.10)	27 (0.34)	122 (1.5)		
Total	651	316 (0.48)	212 (0.33)	136 (0.21)	57 (0.09)	721 (1.11)		

## 4. Discussion

This study was conducted primarily to generate new information about malaria vectors species occurrence, seasonal distribution, and their breeding sites. A total of 721 mosquitoes were collected, all belonging to the genus *Anopheles*, which is the sole mosquito genus capable of transmitting the five human malaria‐causing *Plasmodium* species (*P. vivax*, *P. falciparum*, *P. ovale*, *P. malariae*, and *P. knowlesi*). The predominant *Anopheles* species encountered during the sampling period were identified through morphological characteristics following the keys developed by Gillies and Coetzee [31] were *An. gambiae s.l*, *An. funestus s.l*, *An. coustani*, and *An. pharoensis*. *An. gambiae s.l* was the predominant out of the four species, which accounting for 43.83% (316) of the total. The large number of *An. gambiae s.l* records suggest that this species complex is the predominant and most efficient malaria vector in the study area and its neighboring locations. This finding is consistent with results from a study carried out in the Awassa and Hossana regions during 2006–2007 [22], Bibugn district, Amhara, Ethiopia [32], in Rwanda [33], in Sille, South western part of Ethiopia [34], and in Kenya [35] that *An. gambiae s.l* was the dominant vector in the areas, *An. pharoensis* was recorded as the secondary vector and *An. funestus s.l* was recorded as the third [22, 32]. But the current study was contrasted to the study conducted in southern Ethiopia [34].

The unequal distribution of the malaria vectors species within the area further suggests that, the occurrence of the species truly varies according to macro and micro environmental differences exhibited by different bio‐ecological areas, as was found in studies conducted by Keating et al. [36]. The predominance of *An. gambiae s.l* could be attributed to the adaptability of this species. The other species (i.e., *An. funestus s.l*, *An. coustani*, and *An. pharoensis*) collected occurred in low abundance. *An. funestus* that collected in the dry season were high as compared with others. *An. gambiae* predominantly utilizes temporary pools for breeding; these water bodies become plentiful during the rainy season but usually disappear once the rains stop. In contrast, *An. funestus* tends to reproduce in relatively permanent aquatic habitats that are often covered with vegetation.

The findings of this study demonstrate clear seasonal differences in the proportions of An. *gambiae s.l.* and *An. funestus s.l.* collected, indicating that changes between seasons strongly influence the presence and spatial distribution of malaria vectors in the Shashago area. These variations are likely linked to the distinct habitat preferences of the vector species, whose availability and suitability fluctuate with seasonal environmental conditions.

Our findings differ from those studies conducted in Southwestern Ethiopia [29], where *Anopheles funestus* was the dominant species during the dry season and *An. arabiensis* during the rainy season. In contrast, we found that *An. gambiae s.l.* was the most common species across all study villages and most sampling periods. This difference likely reflects ecological and altitudinal variations between the two study areas. Unlike the lowland and semi‐humid environments studied by Taye and colleagues, the Hadiya Zone lies in a mid‐ to high‐altitude region, where temperature, rainfall, and habitat stability can strongly influence mosquito breeding and survival.

Interestingly, *An. arabiensis* was not found in our collections. This might suggest that the species has been displaced locally or that environmental conditions at higher altitudes are less suitable for its development. These differences highlight how mosquito populations respond to their specific surroundings and why regular, site‐specific monitoring is essential. The contrast with Taye et al. [29] also shows how unique the ecological setting of the Hadiya Zone is reminding us that malaria control strategies need to be adapted to local conditions rather than relying on general regional patterns.

Mosquito breeding occurred throughout the study period in Shashago district, particularly in the selected villages, although the overall density remained relatively low during the dry season. The peak occurrence of malaria vectors during the rainy season is likely attributed to the rainfall, which maintains suitable breeding habitats and supports the development of *Anopheles* larvae. The noticeably high vector abundance highlights the level of nuisance these mosquitoes pose to residents of the study villages and nearby communities. Understanding the factors responsible for seasonal and site‐specific differences in *Anopheles* abundance, as observed in this study, is essential for designing effective control strategies.

A considerable portion of the outdoor‐collected mosquitoes consisted of *An. gambiae s.l.*, *An. funestus s.l.*, *An. coustani*, and *An. pharoensis*. However, some species showed seasonal patterns; for example, *An. funestus s.l.* was mainly detected during the dry season, while others were more common toward the end of the rainy period.

The substantial number of malaria vectors recorded in the surveyed villages corresponds with the high malaria prevalence in the area. This relationship suggests the presence of favorable breeding habitats for *Anopheles* mosquitoes, likely created by freshwater swamps and temporary water pools in farmland areas situated close to houses. The dominant species, *An. gambiae s.l.*, is known to thrive in undisturbed pools formed by river overflow, but does not breed in polluted or alkaline water [37].

The study indicated that most malaria vector species are present across all the surveyed villages, placing all residents at risk of infection. The risk of exposure is influenced by factors such as the nature of people’s occupations and their living conditions, which affect the transmission dynamics of the disease. Malaria transmission is also seasonal, largely driven by rainfall. In October and November, stagnant water provides breeding grounds for mosquitoes, resulting in higher mosquito populations and an increased risk of malaria.

## 5. Conclusions

The present study was an initial effort to investigate the species occurrence and seasonal variation of malaria vectors in the district, with a focus on four selected villages. During the study period, four malaria vector species were identified: *Anopheles gambiae*, *An. funestus, An. coustani, and An. pharoensis.* Among these, *An. gambiae* was the most abundant species, particularly in November, followed by *An. funestus*. *An. gambiae* was consistently collected in large numbers across all seasons and in all study villages. These findings highlight the influence of seasonal fluctuations on the occurrence of different *Anopheles* species and provide valuable information for planning mosquito‐borne disease control strategies in the area.

The study also suggests that larval control could be a cost‐effective approach within integrated vector management if productive habitats are targeted during the dry season when they are geographically limited. To support this, further studies will be conducted to identify productive larval habitats across different environmental settings in the country.

## Funding

No funding was received for this manuscript.

## Consent

The authors have nothing to report.

## Conflicts of Interest

The authors declare no conflicts of interest.

## Data Availability

All the relevant data underlying the results of this study are presented in the manuscript.
